# SMAD2/3 signaling regulates initiation of mouse Wolffian ducts and proximal differentiation in Müllerian ducts

**DOI:** 10.1002/2211-5463.13729

**Published:** 2023-11-20

**Authors:** Tadaaki Nakajima, Akihiro Imai, Chihiro Ishii, Kota Tsuruyama, Risa Yamanaka, Yasuhiro Tomooka, Shinta Saito, Noritaka Adachi, Satomi Kohno, Tomomi Sato

**Affiliations:** ^1^ Department of Biological Science and Technology, Faculty of Industrial Science and Technology Tokyo University of Science Japan; ^2^ Department of Science Yokohama City University Japan; ^3^ Graduate School of Nanobioscience Yokohama City University Japan; ^4^ Department of Biological Sciences St. Cloud State University MN USA

**Keywords:** epithelialization, fate determination, Müllerian duct, TGFβ signaling, Wolffian duct

## Abstract

Male and female reproductive tracts develop from anterior intermediate mesoderm with similar differentiation processes. The anterior intermediate mesoderm develops into the mesonephros, and the Wolffian duct initiates by epithelialization in the mesonephros. The Müllerian duct invaginates from the coelomic epithelium of the cranial mesonephros for ductal formation and is then regionalized into proximal to caudal female reproductive tracts. In this study, we focused on the epithelialization of the Wolffian duct, initiation of the Müllerian duct, and the regionalization step of the Müllerian ducts as a continuous process. By using intermediate mesodermal cells from mouse pluripotent stem cells, we identified that inhibition of SMAD2/3 signaling might be involved in the differentiation into mesenchymal cells, after which mesonephric cells might be then epithelialized during differentiation of the Wolffian duct. Aggregation of coelomic epithelial cells might be related to initiation of the Müllerian duct. Transcriptomic analysis predicted that consensus sequences of SMAD3/4 were enriched among highly expressed genes in the proximal Müllerian duct. SMAD2/3 signaling to regulate differentiation of the Wolffian duct was continuously activated in the proximal Müllerian duct and was involved in proximal and oviductal regionalization. Therefore, SMAD2/3 signaling may be finely tuned to regulate differentiation from initiation to regionalization steps.

## Graphical abstract

We focused on the development of the female reproductive tract from anterior intermediate mesoderm through the Müllerian duct with Wolffian duct as a sequential process. SMAD2/3 signaling to regulate differentiation of the Wolffian duct is involved in proximal and oviductal regionalization. Therefore, SMAD2/3 signaling may be finely tuned to regulate differentiation from the initiation to regionalization step in reproductive tracts.

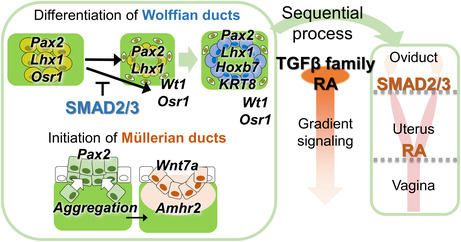

**Abbreviations**
AMH: anti‐Mullerian hormone
AMHR2: AMH receptor 2
BMP: bone morphogenetic protein
E: embryonic day
FGF: fibroblast growth factor
IM: intermediate mesoderm
iPS: induced pluripotent stem
KRT8: keratin 8
Lhx1: LIM homeobox protein 1
MD: Müllerian duct
MEF: mouse embryonic fibroblasts
OSR1: odd‐skipped related transcription factor 1
OVGP1: oviductal glycoprotein 1
PAX2: paired box 2
pSMAD2: phosphorylated SMAD2
RA: retinoic acid
SSR: stem sure serum replacement
T: brachyury
TGFβ: transforming growth factor beta
TUBB4: tubulin beta 4 class
WD: Wolffian duct

Mouse male and female reproductive tracts develop from bilateral anterior intermediate mesoderm (IM). In mesonephros derived from the anterior IM, the pronephric or mesonephric duct, also called the Wolffian duct (WD), forms and elongates toward the caudal region from embryonic day 8.5 (E8.5) to E10.0 [1]. The pronephros degenerates and the mesonephric tubules branch out of the WD and act as fetal kidneys in the cranial region of the mesonephros. The paramesonephric duct, also called the Müllerian duct (MD), is initiated and invaginates into mesenchyme that underlines coelomic epithelium of the specialized cranial mesonephros at E11.5 [2]. MD elongates to the caudal end of the mesonephros along with WD and connects to the urogenital sinus by E13.5. In male mice, the fetal testes secrete testosterone and anti‐Mullerian hormone (AMH). Testosterone maintains WD cells expressing androgen receptor [3], and AMH regresses MD cells expressing AMH receptor 2 (AMHR2) [4]. Since both mesenchymal cells of WD and MD contribute to both stroma of male and female reproductive tracts [5, 6, 7], apparent differences of these mesenchymal cells are little understood, except for androgen receptor and AMHR2 expression. The MD mesenchyme is regionalized into three types of compartments of the oviduct, uterus, and vagina from the proximal to caudal region. After birth, the regionalized mesenchyme irreversibly induces fate determination of those epithelial types [8].

The pronephros in amphibians is induced from animal caps by activin A—a member of the transforming growth factor beta (TGFβ) superfamily—and retinoic acid (RA) [9]. Histological differentiation steps and related transcription factor expression in WD and MD have been revealed *in vivo* [1, 10, 11]. However, the differentiational environment consisting of several factors (e.g. growth factors) for WD and MD initiation is totally unknown in mammals. The mechanism of differentiation of mesonephric cells has been investigated in regenerative studies of kidneys from pluripotent stem cells, because mammalian kidneys consist of metanephros derived from posterior IM expressing brachyury (*T*) and ureteric bud derived from anterior IM expressing odd‐skipped related transcription factor 1 (*Osr1*) [12]. In pluripotent stem cells, regulation of several types of signaling in proper order induces anterior IM cells expressing the specific transcription factors [13, 14, 15, 16] (Fig. 1: E8.5–E10.5) and progesterone‐responsive endometrial stromal fibroblasts [17].

**Fig. 1:**
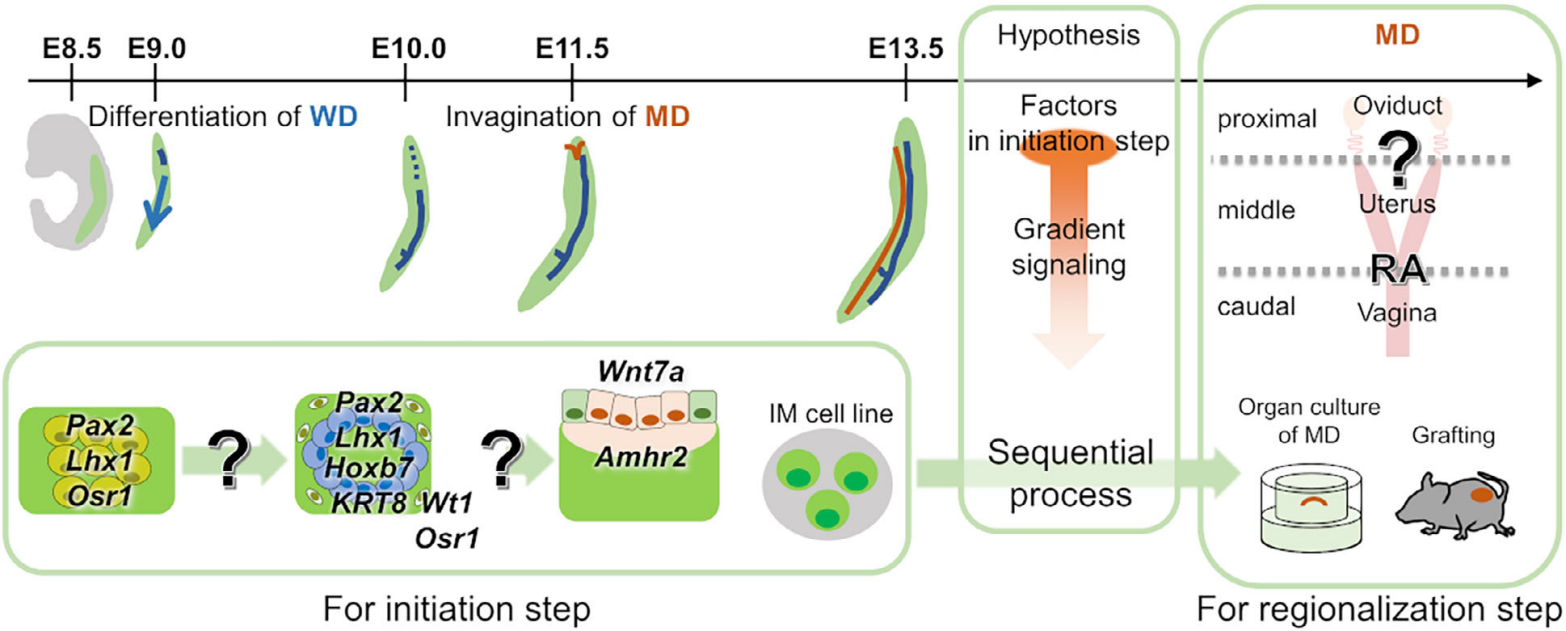
Study design for analyzing development of female reproductive tracts from initiation to the regionalization stage. In this study, an IM cell line was induced from mouse iPS cells to analyze initiation step for WD and MD. Organ culture and a grafting system were used to analyze the regionalizing stage of MD.

Invagination of the mouse coelomic epithelium as a first step for MD development is WD independent [18, 19]. After the initiation step, other cells including mesenchymal cells and WD epithelial cells do not contribute to the MD epithelium [20], while WD is needed for the MD elongation [18]. In avian MD, BMP and FGF signaling induce initiation and invagination of the MD epithelium from the coelomic epithelium under the influence of WD [21, 22]. However, the close contact between WD and MD in mouse development causes inseparability, resulting in that the developmental analysis *in vivo* is difficult due to the low quality and quantity of the sample. In this study, we focused on the WD epithelialization mechanism in the mesonephros and the specialization mechanism for MD initiation in the coelomic epithelium using a simple *in vitro* system.

We hypothesized that some factors involved in differentiation of mesonephros are diverted to initiation and proximal regionalization (irreversible fate determination of mesenchyme) in MD development. In fact, RA signaling is essential for differentiation of the anterior IM remains and determines the border of the uterus and vagina in the MD mesenchyme [23, 24]. Wnt and FGF signaling are needed for mesonephric induction, and Wnt9b and FGF8 are essential for development of mesonephric tubules at cranial mesonephros [19, 25]. In this study, we investigated factors involved in the initiation and regionalization of the development of WD and MD as a continuous process (Fig. 1). Since the WD and MD epithelium and mesenchyme are not completely separated and do not include a large number of cells, we developed a simple induction method of the IM cell line with passaging ability from mouse induced pluripotent stem cells (iPS cells) and analyzed the initiation mechanism using the cell line. Then, we compared the comprehensive gene expression analysis after the initiation process in the MD. As a result, SMAD2/3 signaling was identified as a candidate regulator of proximal differentiation. The effects of SMAD2/3 signaling on MD regionalization were analyzed by organ culture and grafting.

## Materials and methods

### Animals

C57BL/6J for grafting and CD‐1 mice for histology (Sankyo Lab, Tokyo, Japan) were given a commercial diet and tap water *ad libidum* and kept at 22–24 °C under 12 h light/12 h darkness by artificial illumination (lights on 08:00–20:00). The presence of vaginal plugs indicated embryonic day 0.5 (E0.5). The animals were kept according to the NIH Guide for the Care and Use of Laboratory Animals and were approved by the Institutional Animal Care Committee of the Tokyo University of Science (approval number: K15009, K16006, K17007).

### Cell culture

Mouse iPS cells (iPS‐Mef‐Ng‐492B‐4) [26] from RIKEN BRC cell bank were grown on feeder layers of mitomycin C‐treated mouse embryonic fibroblasts (MEF) derived from E14.5 CD1 mice on gelatin‐coated dishes in stem sure DMEM (Wako, Osaka, Japan) supplemented with 15% stem sure serum replacement (SSR; Wako), 1% non‐essential amino acids (NEAA; Wako), 1% Glutamax (Life Technologies, Carlsbad, CA, USA), 0.5 mm 1‐thioglycerol (Sigma‐Aldrich, St. Louis, MO, USA), 1000 units·mL^−1^ leukemia inhibitory factor (Wako), 3 μm CHIR99021 (Cayman Chemical, Ann Arbor, MI, USA), 1 μm PD0325901 (AdooQ Bioscience, Irvine, CA, USA) and 1% penicillin/streptomycin (Nacalai Tesque, Kyoto, Japan) at 37 °C in a humidified 5% CO_2_. For routine passaging, mouse iPS colonies were dissociated enzymatically with 0.25% trypsin and 0.1% EDTA (Sigma‐Aldrich) in PBS.

### Homologous recombination in mouse iPS cells

To establish *Osr1*‐*tdTomato* mouse iPS cells, a *Osr1*‐*tdTomato* targeting vector was developed according to a published method [27] using MultiSite Gateway technology (Thermo Fisher Scientific, Waltham, MA, USA). Briefly, each *Osr1* arm was amplified by genomic PCR with Tks Gflex DNA Polymerase (TaKaRa Bio, Shiga, Japan) and attB‐containing primers (Table S1). Each arm fragment was inserted into each pDONR vector using BP recombination. To add *tdTomato* to the start codon site of the 5′arm, *tdTomato* was cut from the ptdTomato‐C1 vector by AgeI and SmaI and ligated to the 5′arm pDONR vector using AgeI and ScaI. The targeting vectors were made from the pDONR vectors, the pENTR lox‐*Neo* vectors, and the pDEST DTA‐MLS vectors by LR recombination.

After linearization of the targeting vector using I‐SceI, 5 × 10^6^ iPS cells were incubated with 10 μg of the targeting vector in saline including 130 mm NaCl, 5.3 mm KCl, 1.1 mm Na_2_HPO_4_, 1.1 mm NaH_2_PO_4_, 6.1 mm glucose, 0.49 mm MgCl_2_ and 0.9 mm CaCl_2_ at 4 °C for 10 min. These cells were electroporated using a Gene Pulser Xcell (Bio‐Rad Laboratories, Hercules, CA, USA) with an exponential pulse (220 V, 960 μF, 1000 Ω, 4 mm) followed by a transfer pulse (20 V, 50 ms, 10 times, 0.1 s interval). Cells were seeded to MEF fixed with 2.5% glutaraldehyde that retained the ability to maintain the pluripotency of iPS cells for several passages [28]. From the next day, the seeded cells were selected in a medium containing G418. After confirmation of homologous recombination by Southern blotting, iPS cells were electroporated with pBS185 in the above setting to remove of the *Neo* resistance gene using Cre recombinase. Selection was performed using G418.

### Southern blotting

*PGK*‐*Neo* ^*r*^ probes were amplified with Tks Gflex DNA Polymerase and primers (Table S1) and cloned into pMD20 (TaKaRa Bio). The probes of the cloned vector were constructed by PCR with Digoxigenin‐11‐dUTP, alkali‐labile (Roche, Penzberg, Germany). Twenty microgram Genomic DNA isolated from selected iPS clones was digested with EcoT22 I. The genome was separated by electrophoresis on 1% TAE gel. After digestion with 0.5 N NaOH and 1.5 m NaCl and neutralization with 0.5 m Tris–HCl pH 7.5 and 1.5 m NaCl, the gel was blotted to a positively charged nylon membrane with TAE buffer. After UV crosslinking, DNA bands were detected using Amersham Gene Images AlkPhos Direct Labeling Detection System (GE Healthcare, Chicago, IL, USA), anti‐Digoxigenin‐AP, Fab fragments (Sigma‐Aldrich), and CDP‐Star (Sigma‐Aldrich).

### Differentiation protocols in mouse iPS cells

To induce an IM cell line, mouse iPS colonies were dissociated enzymatically and distributed onto 0.1% gelatin‐coated 60‐mm dishes at a density of 4.0 × 10^6^ cells and cultured in DMEM/F12 (Sigma‐Aldrich) supplemented with 10% SSR, 1% penicillin/streptomycin, 1% Glutamax, 1% NEAA, 10 μg·mL^−1^ human insulin (Wako), 100 μg·mL^−1^ human apo‐transferrin (BBI Solutions, Cardiff, UK), 0.05 nm sodium selenite (Sigma‐Aldrich), 0.2 μm putrescine (Sigma‐Aldrich), 0.02 nm progesterone (Sigma‐Aldrich), 0.5 mm 1‐thioglycerol, 3 μm CHIR99021, 10 μm Y27632 (LC Laboratories, New Boston, MA, USA), 1% Matrigel Basement Membrane Matrix (Corning, Durham, NC, USA) and 10 μm retinol (Rol; Sigma‐Aldrich) or RA (Sigma‐Aldrich) (the medium including RA was mentioned as IM medium) at 37 °C in a humidified, 5% CO_2_ incubator. After 2 days, the medium was changed every 3 days. For passaging, the IM cell line was dissociated enzymatically with 0.5% trypsin, 0.2% EDTA, and 0.05% collagenase (Worthington, Lakewood, NJ, USA) in PBS.

To analyze the effects of TGFβ signaling on WD differentiation, 10 ng·mL^−1^ human TGFβ1 (Pepro Tech, Rocky Hill, NJ, USA) or 10 μm SB431542 (Cayman Chemical; a potent inhibitor for TGFβ type I receptor) was added to the IM cell line, 11 days after induction.

To analyze effects of cellular aggregation on MD differentiation, the IM cell line was passaged every 9 days (mentioned as ‘Sparse IM cell line’) or passaged at an over‐confluent state in which aggregation was observed at intervals of 12–18 days (mentioned as ‘Dense IM cell line’). To induce further MD differentiation in the IM cell lines, after the second passaging (at P2), they were cultured in DMEM/F12 supplemented with 2% B27 supplement (Life Technologies), 1% Glutamax, 1% penicillin/streptomycin for 9 days.

### RNA isolation and reverse transcript (RT)‐PCR

Total RNA was isolated from mouse iPSCs, IM cell lines, proximal (upper region from the caudal end of the developing gonad), middle (region from the caudal end of the developing gonads to the fusion point of the ducts), and caudal MD at E14.5 with acid guanidinium‐phenol‐chloroform extraction [29], and reverse‐transcribed into cDNA with ReverTra Ace qPCR RT Master Mix (TOYOBO, Osaka, Japan). An aliquot of cDNA was amplified with specific primers derived from mouse mRNA sequences (Table S2). PCR was carried out with Amplitaq Gold PCR Master Mix (Applied Biosystems, Foster City, CA, USA). For semi‐quantitative analysis, images of electrophoretic gels of PCR products were analyzed using imagej (National Institutes of Health, Bethesda, MD, USA). Quantitative RT‐PCR was performed using the ABI Prism 7000 detection system (Applied Biosystems) with KOD SYBR qPCR Mix (TOYOBO) according to the manufacturer's protocol. *Β‐actin* was chosen as an internal standard. Ten Müllerian ducts were pooled in one sample. Three biologically independent experiments were carried out.

### RNA sequencing (RNA‐seq)

Total RNAs of the proximal and middle MD at E14.5 were extracted from 10 mice with a Total RNA Isolation System (Promega, Madison, WI, USA). A cDNA library was constructed with a TruSeq Stranded Total RNA (Illumina, San Diego, CA, USA) and sequenced with a single‐end Illumina HiSeq 2500 (Illumina) (*n* = 1). Raw data were annotated to mouse genes (NCBIM37 from ucsc: http://genome.ucsc.edu/index.html). Enrichment analysis for consensus sequences in the promoter region was performed using pscan (http://159.149.160.88/pscan/). GO terms and KEGG pathway analysis were performed using david (https://david.ncifcrf.gov/).

### Fluorescence‐activating cell sorting (FACS) analysis

*Osr1*‐*tdTomato* iPS colonies were detached with 2.5% trypsin‐1% EDTA solution at 37 °C for 4 min. To remove feeder cells, the cell suspension was cultured in gelatin‐coated dishes at 37 °C for 40 min and the attached cells were removed. Floating iPS cells were harvested with PBS. IM cell lines at passages 1 and 5 were detached with 2.5% trypsin‐1% EDTA solution at 37 °C for 10 min and harvested with PBS. These cells were analyzed for *Nanog*‐EGFP and *Osr1*‐tdTOMATO by Cell Sorter SH800 (SONY, Tokyo, Japan).

### Organ culture and grafting

Eight volumes of Cellmatrix type I‐A (Nitta Gelatin, Osaka, Japan) were mixed with 1 volume 10 × DMEM/F12, and then 1 volume 200 mm HEPES buffer containing 262 mm NaHCO_3_ and 0.05 N NaOH was added to the mixture. The 350‐μL cold collagen mixture was poured into cell culture inserts (Corning) placed in wells of a 24‐well plate and allowed to gel at 37 °C for 30 min. Mesonephros at E11.5 and MDs at E13.5 were collected and placed in Hanks buffer (Wako). The tissues were washed three times with Hanks buffer and mixed with 350 μL of fresh and cold collagen mixture. The mixture was overlaid on the base of collagen gel in each cell culture insert and allowed to gel at 37 °C for 30 min. Subsequently, 200 μL 20% fetal bovine serum (Cell Culture Technologies, Zurich, Switzerland) in DMEM/F12 were added to each well and tissues were cultured at 37 °C in a humidified, 5% CO_2_ incubator for 7 days with daily medium changes. Agents added to the medium were 10 μm RA, 10 μm AGN193109 (Toronto Research Chemicals, Toronto, Canada), 10 ng·mL^−1^ mouse activin A (R&D Systems, Minneapolis, MN, USA) and 10 μm SB431542 (Cayman Chemical). Four to 8 MDs were grafted under the renal capsules of 2‐month‐old ovariectomized female C57BL/6J mice. For female reproductive tracts, the grafts were harvested at 30 days post‐grafting. Three to five independent grafting experiments were carried out. After immunofluorescence, the graft ducts were counted to reveal the number of biological experimental replication (*n*).

### Histological analyses

Embryos from E8.5 to E11.5 were fixed in 4% paraformaldehyde (PFA) at 4 °C overnight. The embryos at the other developmental stage were eliminated by microscopic visual confirmation. Samples were dehydrated with a graded alcohol series, embedded in paraffin, and cut into 6‐μm sections. Sections were deparaffinized with xylene, rehydrated, and stained with hematoxylin and eosin (HE).

### Immunofluorescence

Whole bodies at E8.5 and 9.5, lower bodies without livers and guts from E10.0 to E14.5, and MD‐grafts were fixed in 4% PFA at 4 °C for 24 h. Samples were dehydrated with graded alcohol series and embedded in paraffin and cut into 6‐μm sections. For antigen retrieval on paired box 2 (PAX2), vimentin, keratin 8 (KRT8), tubulin beta 4 class (TUBB4), and phosphorylated SMAD2 (pSMAD2) staining, sections were immersed in 0.05% sodium citraconic acid (pH 7.4) at 95 °C for 45 min. Nonspecific binding was blocked in PBS containing 5% goat serum and 1% bovine serum albumin (BSA) for 30 min at room temperature. Sections were incubated at 4 °C overnight with primary antibody for PAX2 (1/100; Invitrogen, Carlsbad, CA, USA), KRT8 (1/20; Progen Biotechnik GmbH, Heidelberg, Germany), vimentin (1/100; Santa Cruz Biotechnology, Dallas, TX, USA), laminin (1/500; LSL, Tokyo, Japan) oviductal glycoprotein 1 (OVGP1; 1/200; Santa Cruz Biotechnology), TUBB4 (1/200; Boehringer Mannheim Biochemica, Mannheim, Germany) in blocking reagent or pSMAD2 (1/50; Cell Signaling Technology, Danvers, MA, USA) in blocking reagent containing 0.1% Triton X‐100. After washing with PBS containing 0.1% Tween 20, the sections were incubated with Alexa Fluor 488‐ or 594‐conjugated anti‐mouse or rabbit IgG goat antibody (1/250; Jackson Immuno Research, West Grove, PA, USA) at room temperature for 2 h. For negative controls, normal rabbit IgG (Santa Cruz Biotechnology) was used. 4′, 6‐diamino‐2‐phenylindole (DAPI) was used to stain nucleic acids. Fluorescence intensity of pSMAD2 staining in the nucleus was quantified only in the DAPI‐positive region using imagej. For quantification, the comparison samples were stained at the same time.

### Statistical analysis

All data were expressed as means ± standard errors. Two‐tailed Student *t*‐test or Welch *t*‐test was used for single comparisons. For multiple comparisons, differences were estimated using a one‐way Peritz, Games‐Howell, Dunnett's step‐down test. A statistically significant difference was defined as *P* ≤ 0.05.

## Results

### WD epithelialization mechanism through mesenchymal‐epithelial transition (MET)

**Fig. 2:**
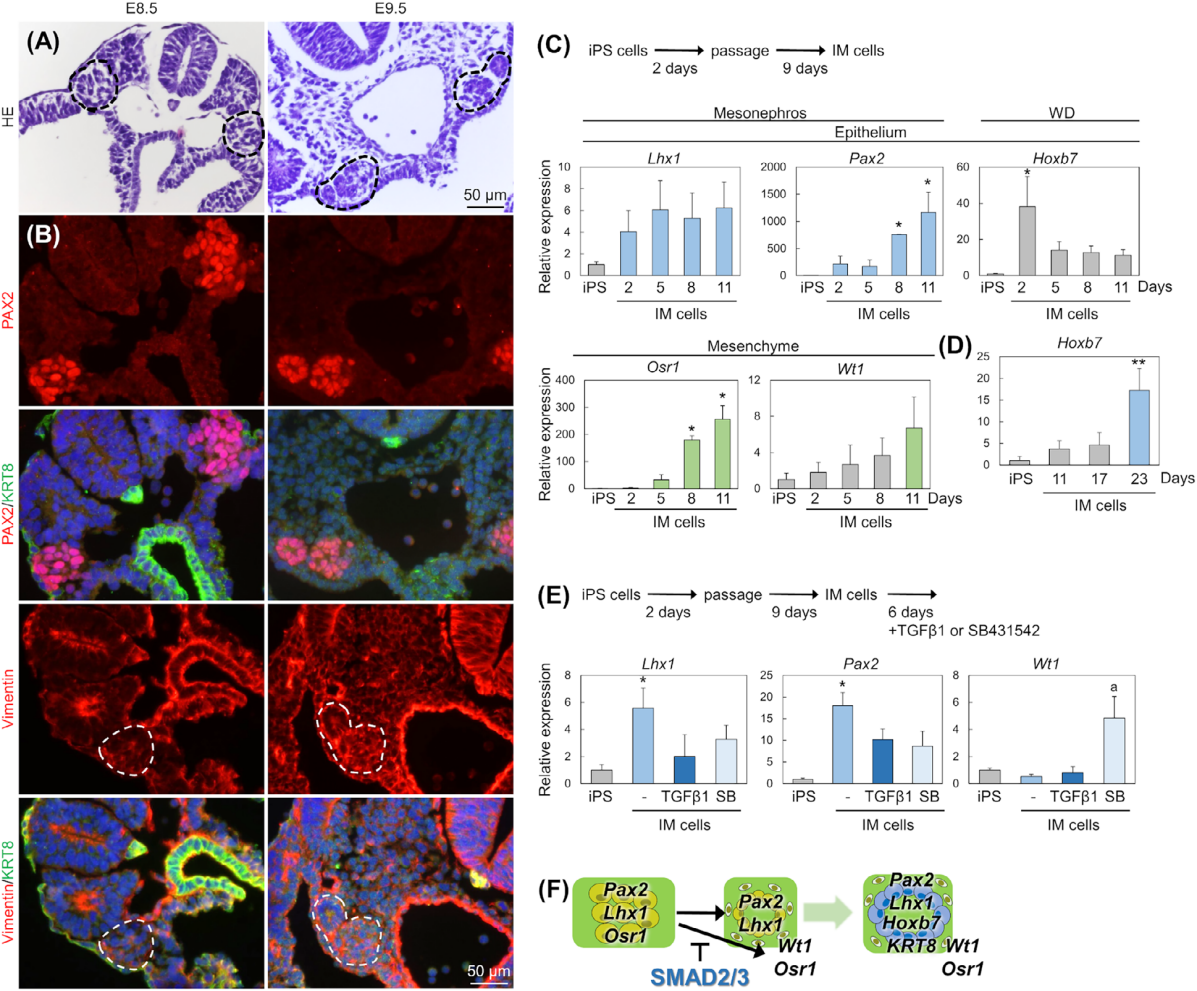
Differentiation of epithelium and mesenchyme of WD from the mesonephros. (A) Histology of a cross‐section of the mesonephros in E8.5 and E9.5 embryos with HE staining. Dashed line region: mesonephros. (B) PAX2 (early mesonephric marker, red) or vimentin (mesenchymal cytoskeleton, red) and KRT8 (epithelial cytoskeleton, green) expression in serial sections of the E8.5 and E9.5 mesonephros (*n* = 3). Blue: nuclei. Dashed line region: mesonephros. Scale bar = 50 μm. (C) Mesonephric and WD epithelial and mesenchymal marker gene expression in the differentiation process of IM cells from iPS cells was analyzed by semi‐quantitative RT‐PCR (the raw gel images were listed in Fig. S4; *n* = 3). Data were expressed relative to mRNA expression of iPS cells (=1.0). *: *P* ≤ 0.05 with that in iPS cells by Dunnett's step‐down test. (D) *Hoxb7* expression in the late differentiation process of IM cells from iPS cells was analyzed with quantitative RT‐PCR (*n* = 3). Data were expressed relative to mRNA expression of iPS cells (=1.0). **: *P* ≤ 0.05 with that in other cells by Peritz or Games‐Howell test. (E) Effects of TGFβ1 and TGFβ signal inhibitor, SB431542 (SB), on mesonephric epithelial and mesenchymal marker gene expression in IM cells with semi‐quantitative RT‐PCR (the raw gel images were listed in Fig. S4). *: *P* ≤ 0.05 with that in iPS cells. a: *P* ≤ 0.05 with that in non‐treated IM cells by Peritz or Games‐Howell test. The error bars represent SEM. (F) A model image of WD epithelialization.

We first analyzed the epithelialization mechanism in the mesonephros histologically *in vivo* to redefine the cellular dynamics of WD differentiation. The duct‐like shape of WD in the mesonephros was already observed in E8.5 of the mesonephros (Fig. 2A). At this stage, PAX2 (early mesonephric epithelial marker) were expressed in almost all cells of the mesonephros, and vimentin (mesenchymal cytoskeleton) was weakly expressed, but KRT8 (epithelial cytoskeleton) was not expressed (Fig. 2B). At E9.5, PAX2 and vimentin were continuously expressed in the tubular structure (vimentin expression was especially detected at the basal region of the cells). These results suggested that the phenotype of mesonephric epithelial cells expressing PAX2 is similar to the mesenchymal type and that differentiation of MET is needed for WD epithelialization.

To confirm the time course of WD developmental *in vitro* for analysis of the factors involved in WD differentiation, the expression profiles of marker genes were traced in the induction of intermediate mesodermal cells from iPS cells by the addition of several factors (serum, supplements, activators for Wnt, BMP, FGF, and RA signaling) and combinations thereof with several time courses (Fig. 2C). LIM homeobox protein 1 (*Lhx1*), *Pax2*, and *Osr1* are expressed from the IM to the pronephros and the epithelium of the mesonephros, and the pronephros is very rudimentary in the mouse [1]. Therefore, we used *Lhx1* and *Pax2* for IM and mesonephric epithelial markers. *Osr1* is coexpressed with *Lhx1* and *Pax2* at an early stage [15, 30], essential for the formation and maintenance of the mesonephric duct, and restricted in the mesenchyme from E9.5 [31, 32]. In this induction process for the IM cell line, we confirmed early induction of *Lhx1*, *Pax2*, and *Osr1* expression. *Wt1* (mesonephric mesenchymal marker) was induced in IM cells after 11 days. The increase in *Hoxb7* at day 2 was unexpected; however, the expression was immediately downregulated on day 5, suggesting that *Hoxb7* was not stably increased at this stage. The induction of *Hoxb7* was detected at 23 days (Fig. 2D). Taken together, early‐stage mesonephros consisting of *Lhx1*‐, *Pax2*‐, and *Osr1*‐expressing mesonephric epithelial cells differentiated into WD epithelial and mesenchymal cells (Fig. 2F).

We investigated that TGFβ signaling (a well‐known inducer of EMT, and important for differentiation of mesonephros) is involved in differentiation of *Wt1*‐expressing WD mesenchymal cells from mesonephric epithelial cells *in vitro* model. Treatment with TGFβ1 did not significantly affect *Lhx1*, *Pax2*, and *Wt1* expression (Fig. 2E). This result was consistent with the mesenchymal phenotype of mesonephric epithelial cells (Fig. 2A). However, inhibition of SMAD2/3 signaling by SB431542 increased *Wt1* expression. Therefore, inhibition of TGFβ signaling may be required for induction of WD mesenchymal cells from mesonephros (Fig. 2F).

### Specification mechanism of the initiation site for MD at the coelomic epithelium

**Fig. 3:**
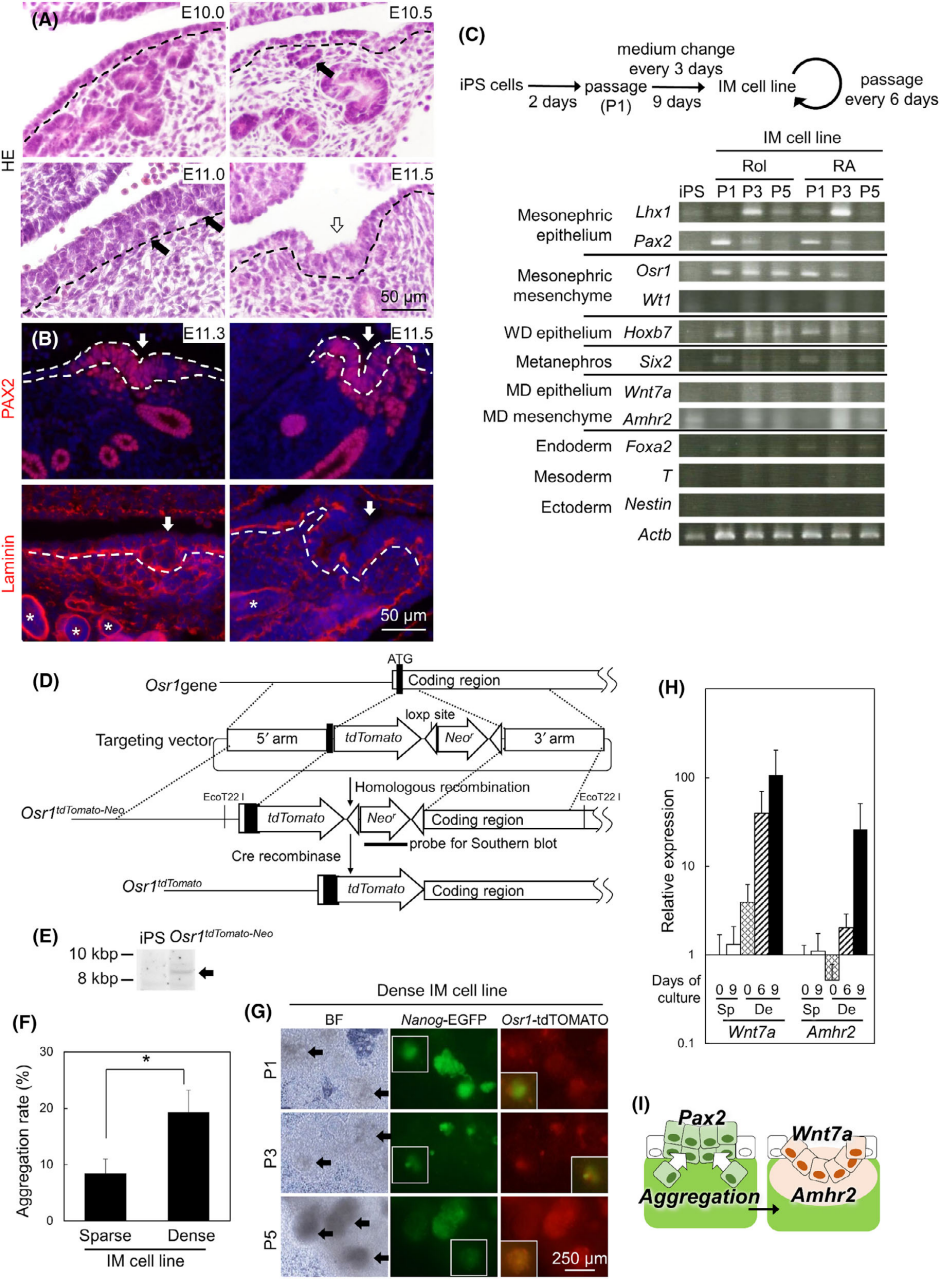
Differentiation of epithelium and mesenchyme of MD from an IM cell line. (A) Histology of a sagittal section of an anterior mesonephros in E10.0 and E11.5 embryos with HE staining. Dashed line: coelomic epithelium. Black arrows: degenerated tubules. White arrow: invagination point. (B) PAX2 (mesonephric epithelial marker, red) or laminin (basement membrane, red) expression at the invagination point of the mesonephros in E11.3 and E11.5 embryos (*n* = 5) Blue: nuclei. *: tubule structures. Scale bar = 50 μm. (C) Expression profiles for differentiation marker genes in IM cells established with retinol (Rol) or retinoic acid (RA) treatment (*n* = 4; the independently induced samples from iPS cells). P: passage number samples in the same line. (D) A method of gene targeting to produce *Osr1*‐*tdTomato* mouse iPS cells. (E) A Southern blot analysis using a PGK‐*Neo* ^*r*^ probe to confirm homologous recombination. Arrow: ca. 8.6‐kbp band after digestion with EcoT22 I (the raw image was listed in Fig. S4). (F) The rate of aggregation in the fields of images in sparse and dense IM cell lines (*n* = 3). *: *P* ≤ 0.05 by two‐tailed Student *t*‐test. (G) Bright field (BF) and *Nanog*‐EGFP, or *Osr1*‐tdTOMATO fluorescent images in the dense IM cell line from passage number 1 (P1) to P5. Arrows: aggregation. White squares in red fluorescent images: merge images at white squares in green fluorescent images. Scale bar = 250 μm. (H) *Wnt7a* (MD epithelial marker) and *Amhr2* (MD mesenchymal marker) expression in the sparse (Sp) and dense (De) IM cell lines at P2 from day 0 to 9 of culture was analyzed by quantitative PCR (*n* = 3). Data are expressed relative to mRNA expression of the sparse IM cell line at day 0 (=1.0). The error bars represent SEM. (I) A model image of MD initiation.

The MD initiation step was also analyzed in detail with histology *in vivo* (Fig. 3A). At the cranial region of the mesonephros at E10.0, the coelomic epithelium is almost one layer and the tubular structures were situated near the coelomic epithelium. Thickness of the coelomic epithelium increased from E10.0 to E11.0. In the near region of the coelomic epithelium, tubular structures were detected at E10.0–E10.5 and may be contained in the thickened coelomic epithelium at E11.0. At E11.5, the thickening epithelium invaginated to the mesenchyme. We revealed the situation of tubular epithelial cells around the initiation site of the coelomic epithelium with immunofluorescence for PAX2 and laminin (Fig. 3B). At the starting point of invagination at E11.3, PAX2‐expressing cells were detected not only in the thickening coelomic epithelium, but also in the mesenchyme surrounding the initiation site. Laminin (a component of basement membrane) expression expanded to mesenchyme at the E11.3 initiation site. At E11.5, PAX2‐expressing cells in the mesenchyme were detected at the invagination site, and the laminin expression at the mesenchyme was weakened. At the tip of invagination, the laminin‐expressing border between epithelium and mesenchyme was disrupted the dot‐like laminin expression in the basement membrane of tip region. This aggregation of the initiation site of MD may be important for differentiation into MD.

For long‐term culture of IM cells from iPS cells to induce MD differentiation *in vitro*, we tried to passage the IM cells in the culture condition. Under conditions with activation of Wnt and RA signaling, we found mixed clusters with most differentiated *Nanog*‐negative cells strongly expressing IM marker genes and weakly expressing other tissue marker genes, and a small number of *Nanog*‐positive cells. Thus, we hypothesized that the low population of pluripotent cells was maintained and IM cells were continuously induced under stable conditions. The modulated condition induced an IM cell line that possessed the passaging ability at least five times from iPS cells (Fig. 3C). In IM cell lines, *Lhx1*, *Pax2*, *Osr1*, *Hoxb7*, and *Six2* expression was detected with low expression of *Foxa2*, *T*, and *Nestin*, suggesting that differentiation into IM was induced in IM cell lines. *Wt1*, *Wnt7a* and *Amhr2* expression is low or unstable in the IM cell line. The RA treatment can be replaced with Rol, which is the precursor of RA, indicating RA‐synthesizing ability in the IM cells.

In the induction culture of the IM cell line, we had found that passaging before formation of cellular aggregation invariably failed to increase of *Wnt7a* and *Amhr2* expression. To trace the differentiation of IM in sparse culture with early passaging and dense culture with cellular aggregation, *tdTomato* reporter was inserted into the *Osr1* gene in *Nanog*‐EGFP iPS cells with homologous recombination (Fig. 3D). Homologous recombination was confirmed by Southern blotting with the *PGK*‐*Neo* probe at 8.6 kbp (Fig. 3E). To analyze the effects of cellular aggregation on differentiation of IM, the IM cell line was induced from *Osr1*‐*tdTomato* iPS cells. The sparse IM cell line was passaged before reaching a confluent state and the dense IM cell line was passaged at an over‐confluent state. In the dense IM cell line, the cellular aggregation rate was higher than that in the sparse line (Fig. 3F). *Nanog*‐EGFP and *Osr1*‐tdTOMATO were expressed at neighboring sites of the same aggregates in dense IM cell lines from passage number 1 (P1) to P5 (Fig. 3G). Although quantification of these populations is difficult by FACS due to the lower expression of tdTOMATO than EGFP and the slight overlap excitation and emission, EGFP‐positive cells were apparently decreased, and tdTOMATO‐positive cells may be increased in dense IM cell lines (Fig. S1). The sparse IM cell lines did not proliferate sufficiently from P2 and could not be passaged by P5. Thus, aggregation is important for induction of the IM cell line. Furthermore, *Wnt7a* (MD epithelial marker) and *Amhr2* (MD mesenchymal marker that is induced by Wnt7a [4]) expression were analyzed in sparse and dense IM cell lines at P2. In the dense IM cell lines only, *Wnt7a* and *Amhr2* tended to increase at 6 and 9 days, respectively (Fig. 3H). These data provide the possibility that stacking epithelial cells may be important for specification of the MD epithelium from mesonephros (Fig. 3I).

### Candidate signaling for proximal regionalization in MD

**Fig. 4:**
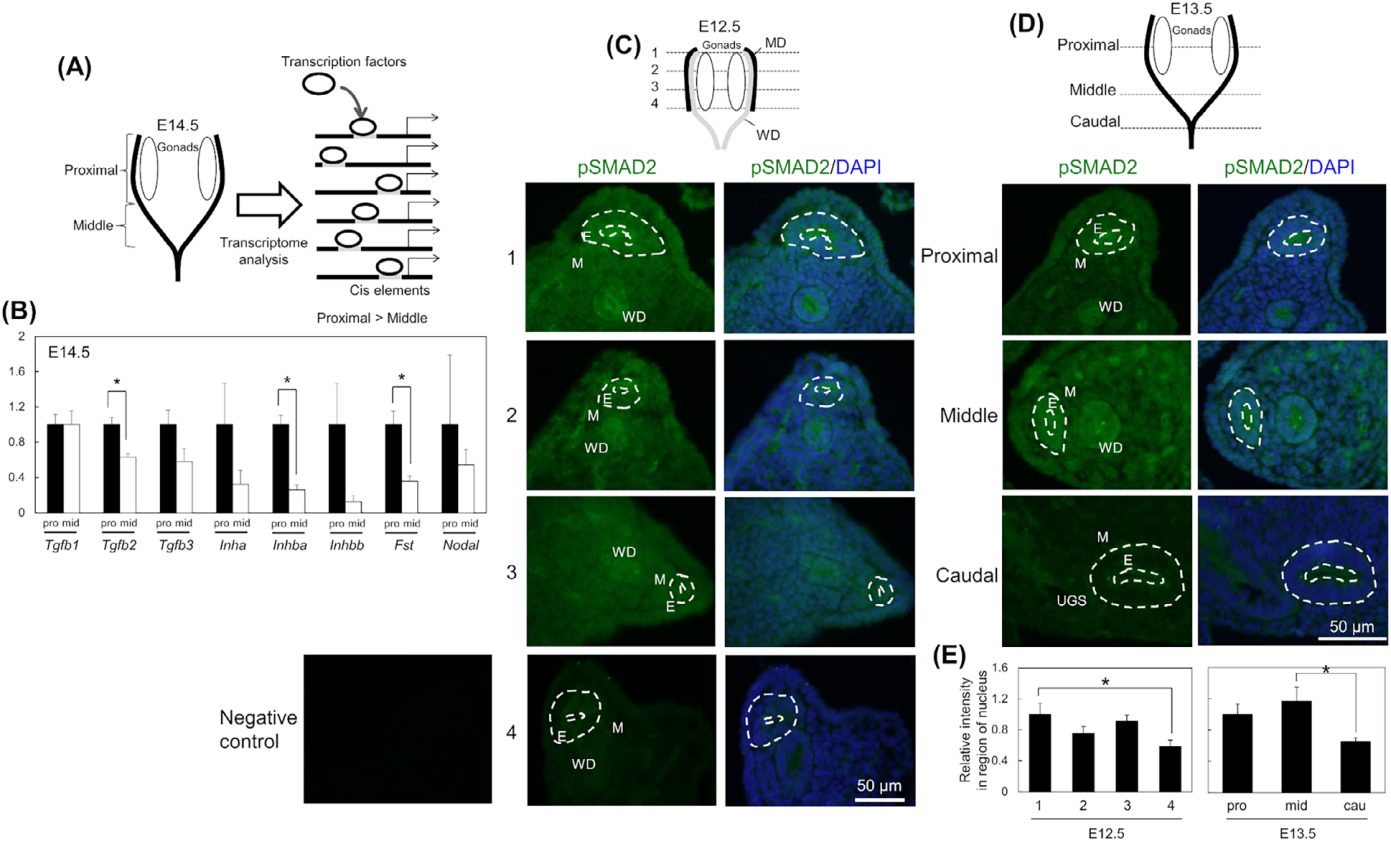
An attempt to identify candidate signaling related to proximal differentiation in MD. (A) A schematic image of transcriptomic analysis in an attempt to identify candidate transcription factors regulating proximal and oviductal development. Proximal and middle MDs at E14.5 were separated at the bottom of the gonads. After RNA‐seq (*n* = 1), 2 or 3‐fold higher gene expression in the proximal and middle region was observed. Transcription factors were predicted as binding to significantly accumulated consensus sequences in promoter regions of the gene lists (File S1). (B) Region‐specific expression of *Tgfb1*, *Tgfb2*, *Tgfb3*, *Inha*, *Inhba*, *Inhbb*, *Fst*, and *Nodal* mRNA was analyzed in proximal (pro) and middle MDs (mid) at E14.5 by quantitative PCR (*n* = 3). Data are expressed relative to mRNA expression of the proximal MD (=1.0). *: *P* ≤ 0.05 by two‐tailed Student *t*‐test or Welch *t*‐test. (C, D) Localization of pSMAD2 and merged images with DAPI staining from proximal to caudal regions of MDs at E12.5 (C, *n* = 3) and E14.5 (D, *n* = 4). Positions of cross‐sections are indicated in the top images. Green: pSMAD2‐positive cells. Blue: nuclei. White dashed line: borderline of MD epithelium. E, epithelium; M, mesenchyme. Scale bar = 50 μm. (E) The pSMAD2 intensity in the nucleus was quantified in DAPI‐positive nuclei in the proximal (pro), middle (mid) and caudal regions (cau). *: *P* ≤ 0.05 by Peritz test. The error bars represent SEM.

Next, we compared factors for differentiation of mesonephros and comprehensive gene expression analysis to identify candidates for proximal regionalization in MD. Comprehensive gene expression in proximal and middle MD at E14.5 was detected by RNA‐seq (Fig. 4A; This data was estimation because of *n* = 1). For a search for regulative signaling, enrichment of consensus sequences for transcription factors was analyzed in 2‐ or 3‐fold higher genes in each proximal or middle MD. The consensus sequences for SMAD3/4 and WT1 were enriched in the proximal and middle MD, respectively (File S1). The analysis of the GO term and the KEGG pathway showed that high expression of *Hox* genes was confirmed in the middle MD. Further, the accumulation of extracellular matrix genes in the proximal MD and genes related to BMP and genes related to the nervous system in the middle MD were newly found. We focused on SMAD2/3 signaling as diverted signaling from earlier development because activins and TGFβ regulate differentiation of IM and WD (e.g., Fig. 2). In expression profiles of ligands that can activate SMAD2/3 signaling, expression of all ligands was predictably high or equal in the proximal MD than that in the middle MD (Fig. 4B).

To detect the activation of SMAD signal only in the region of MDs, immunofluorescence was chosen because the protein extraction of only MDs is hard at the developmental stages. the high expression of pSMAD2 (activated SMAD2) was detected at the proximal MD at E12.5 when the MD was elongating (Fig. 4C) and E13.5 when elongation of MD reached the urogenital sinus (Fig. 4D). In the quantitative analysis of pSMAD2 expression only in the nuclei, the intensity of pSMAD2 signal in the proximal region was significantly higher than that in the caudal region at E12.5 and E13.5 (Fig. 4E).

**Fig. 5:**
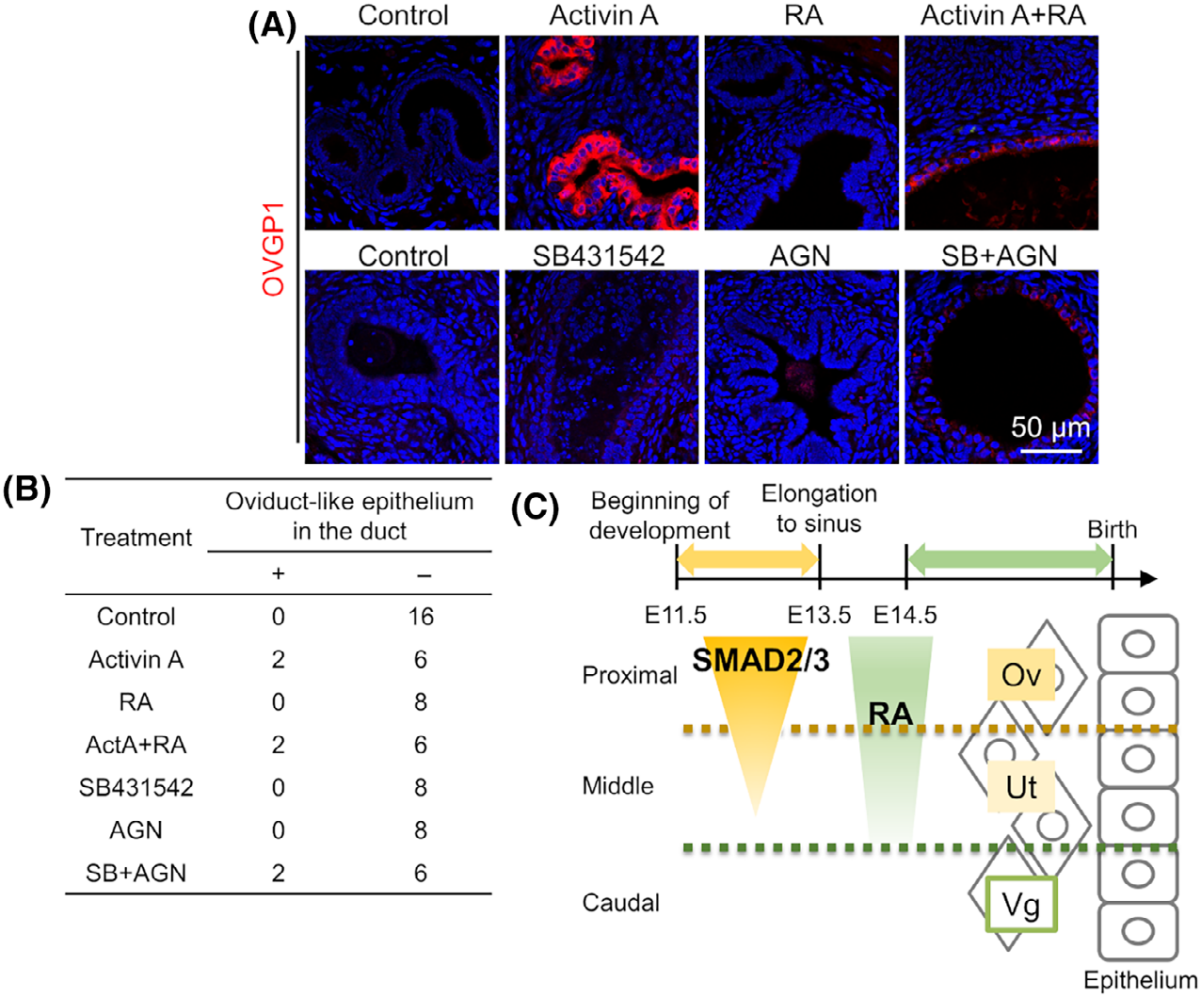
The role of SMAD2/3 signaling in proximal differentiation of MD. E11.5 MDs were cultured with activin A, SB431542 (SB), RA, and/or AGN193109 (AGN) for 7 days, and organ‐cultured MDs were then grafted for 30 days. (A) OVGP1 (oviductal secretory epithelial marker) expression in grafted MDs (*n* = 8 or 16). Scale bar = 50 μm. (B) A table of ducts having oviduct‐like epithelium (ovi) containing OVGP1‐expressing cells in grafted MDs. (C) A schematic image of proximal fate determination in MD.

RA can induce regionalization into upper MD in the organ culture system and the fate is irreversible even after induction of further differentiation *in vivo* grafting [23]. Using the method, to determine the effects of SMAD2/3 signaling on irreversible proximal fate determination, the organ‐cultured MD at E13.5 with activin A and SMAD2/3 signal inhibitor was grafted under the renal capsule to induce differentiation of oviduct and uterus; however, no effect on differentiation of proximal MD was detected by activation or inhibition of SMAD2/3 signaling with regulation of RA signaling (Fig. S2). Considering that irreversible determination of proximal fate may already have begun at elongation stage, the mesonephros at E11.5 was used for this experiment. Activation and inhibition in pSMAD2 were confirmed in the organ culture with activin A and SB431542, respectively (Fig. S3). After grafting, the expression of OVGP1 (marker of oviductal secretory epithelial cells) was detected in organ‐cultured MD treated with activin A, activinA + RA, and inhibitor of SMAD2/3 + RA signaling, but not in control MD (Fig. 5A,B). The two reports of OVGP1 expression in each treatment group were found in the different grafts. Therefore, the high expression of ligands and activation of SMAD2 in the proximal region of the elongated MD were involved in the proximal and oviductal regionalization (Fig. 5C).

## Discussion

In this study, we induced an IM cell line from pluripotent stem cells and proposed some insights into the initiation mechanism of WD and MD using the IM cell line. The culture system to induce specific mesonephric cells has been established with complex methods [14, 15]. Further, metanephric progenitors induced from pluripotent stem cells can be maintained under culture conditions [33]. However, no method has been reported for the maintenance of mesonephric cells. The IM cell line induced with a simple method can be passaged, although mesonephric cells may not be directly maintained, and the *Nanog*‐positive cluster may continuously differentiate to IM cells under these conditions.

In the IM cell line, it seemed that mesonephric differentiation from pluripotent stem cells was continuously induced in stable condition with activation of Wnt and RA signaling. For mesonephric induction, the activation of Wnt, RA, and FGF signaling of a suitable duration is consistent in several methods [14, 15, 17, 30]. However, if specific IM cell types are not needed, IM cells can be induced without control of time course and intensity for Wnt and RA signaling and addition of the FGF signaling activator. FGF ligands were expressed in the posterior region of IM in chickens, and FGFs stimulate the WD epithelialization [34]. Furthermore, some types of cells in the IM cell line have the ability to synthesize RA from Rol. Wnt, RA, FGF, and other ligands can be supplied and regulated by secretions from cells under these culture conditions because several types of IM cells were mixed in the IM cell line.

In the mesonephros at an early stage, the epithelial and mesenchymal cell types were not distinguishable because the entire mesonephros consisted of PAX2‐expressing cells that possess a mesenchymal phenotype. PAX2‐ and vimentin‐expressing cells may convert to *Hoxb7*‐ and KRT8‐expressing WD epithelial cells with MET. FGF8 signaling acts in WD epithelialization in *Xenopus laevis* and chickens [34, 35]. We revealed that inhibition of TGFβ signaling was one of the regulators for WD mesenchymal differentiation. An inhibitor for TGFβ signaling is used in some methods for induction of WD from pluripotent stem cells [15, 30]. Inhibition may stimulate *Wt1* expression and WD mesenchymal differentiation.

In histological analysis of MD, remnants of degenerated tubules may be contained in the thickening coelomic epithelium and PAX2‐expressing cells were detected not only in the thickening coelomic epithelium, but also in the mesenchyme surrounding the initiation site. At the invagination site, the laminin‐expressing border between the epithelium and mesenchyme was disrupted. Laminin expression is lost at the MD invagination site in avians [22]. These preliminary data led us to hypothesize that degenerate pro/mesonephric tubules expressing PAX2 could contribute to the coelomic epithelium through the disrupted basement membrane, and the thickened coelomic epithelium containing PAX2‐expressing cells formed the initiation site of MD. In the future, lineage tracing experiments will be need using markers for pro/mesonephric tubules.

MD epithelial cells were induced with cellular aggregation in dense IM cell lines. Physical force such as compression pressure changes the conformation of actin fibers in the cytoplasm and regulates Hippo signaling in the nuclei [36]. Aggregation and change in cell–cell junctions regulate canonical Wnt signaling [37, 38, 39]. Further, canonical Wnt signaling is activated at the initiation site of MD [40]. These reports suggest that signaling changes by aggregation or compression may trigger the specification of the MD initiation site from the coelomic epithelium. PAX2‐ and laminin‐expressing cells and degenerated ducts were situated surrounding the mesenchyme at the initiation site. The histological data provides one possibility that degenerated PAX2‐expressing nephric epithelial cells can convert to other nephric epithelial cells. For one example of conversion, in zebrafish in which a mesonephros acts as a mature kidney, the pronephric ducts are not completely degenerated, and they are converted to the collecting duct [41]. Thus, degenerated pronephric ducts or mesonephric tubules at cranial mesonephros might be converted to aggregated epithelium of the initiation site of MD.

## Conclusions

We tried to consider the differentiation of mesonephros and MD as a continuous process and found that SMAD2/3 signaling is involved in WD and MD from initiation to regionalization stages. RA signaling related factors may form a gradient by cell proliferation during elongation [23, 24], and ligands and SMAD2/3 signaling can be similarly decreased by elongation of MD. Thus, MD regionalization may be mainly caused by differences in the elongation mechanism. In the transcriptomic analysis in E14.5 MD, genes related to the extracellular matrix (ECM, e.g., Tenascin) or the nervous system were high in the proximal or middle region, respectively. These ECMs are involved in the formation of mesodermal regenerative blastema in the skeletal muscle of the limb and heart [42, 43]. The uterus is a highly innervated organ [44], and in a model for endometriosis, the ectopically transplanted uterus induces innervation [45]. Therefore, an environment of ECM and innervation may be involved in differentiation of the oviduct and uterus. RA and SMAD2/3 signaling were not sufficient for proximal regionalization of MD, because regulation of these signaling types induced almost none of the transplants consisting of only oviducts. Transcriptomic analysis showed gene enrichment relating to BMP signaling that is necessary for mesonephric induction. In the future, other signaling types for regionalization will be identified.

## Conflict of interest

The authors declare no conflict of interest.

### Peer review

The peer review history for this article is available at https://www.webofscience.com/api/gateway/wos/peer‐review/10.1002/2211‐5463.13729.

## Author contributions

TN contributed to conceptualization, funding acquisition, project administration, supervision, formal analysis, investigation, methodology, validation, visualization, and writing of the manuscript. AI, CI, KT, RY participated in data curation, investigation, methodology, validation, visualization, and writing, reviewing, and editing the manuscript. YT contributed to funding acquisition, project administration, resources, supervision, and writing, reviewing, and editing the manuscript. SS, NA, SK supported investigation, methodology, and writing, reviewing, and editing the manuscript. TS contributed this study at conceptualization, resources and writing, reviewing, and editing the manuscript.

## Supporting information


**Fig. S1.** The population of pluripotent and differentiated cells in the IM cell line.
**Fig. S2.** The effect of activation of SMAD2/3 signaling in MD at E13.5.
**Fig. S3.** SMAD2 activation in organ‐cultured MDs.
**Fig. S4.** The raw gel images.
**Table S1.** Primers for homologous recombination.
**Table S2.** Primers for RT‐PCR.

**File S1.** RNA‐seq data in the proximal and middle MD at E14.5.

## Data Availability

RNA‐Seq data were uploaded as Supplemental data.
