# Supplementary material for: SMAD2/3 signaling regulates initiation of mouse Wolffian ducts and proximal differentiation in Müllerian ducts
Source: FEBS Open Bio. 2023 Nov 20;14(1):37–50. doi: 10.1002/2211-5463.13729 (PMC10761927; doi:10.1002/2211-5463.13729)
Supplement: Supplementary file 1 — Fig. S1. The population of pluripotent and differentiated cells in the IM cell line. Fig. S2. The effect of activation of SMAD2/3 signaling in MD at E13.5. Fig. S3. SMAD2 activation in organ‐cultured MDs. Fig. S4. The raw gel images. Table S1. Primers for homologous recombination. Table S2. Primers for RT‐PCR. [file FEB4-14-37-s001.pdf]

Title: SMAD2/3 signaling for initiation of mouse Wolffian ducts is diverted to proximal differentiation in mouse Müllerian ducts

Authors: Tadaaki Nakajima[1, 2*], Akihiro Imai[1], Chihiro Ishii[1], Kota Tsuruyama[1], Risa Yamanaka[1], Yasuhiro Tomooka[1], Shinta Saito[2], Noritaka Adachi[2, 4], Satomi Kohno[3], Tomomi Sato[2, 4].

1: Department of Biological Science and Technology, Faculty of Industrial Science and Technology, Tokyo University of Science, 6-3-1 Niijuku, Katsushika-ku, Tokyo 125-8585, Japan.

2: Department of Science, Yokohama City University, 22-2 Seto, Kanazawa-ku, Yokohama 236-0027, Japan.

3: Department of Biological Sciences, St. Cloud State University, 720 4th Ave S, St. Cloud, MN 56301, USA.

4: Graduate School of Nanobioscience, Yokohama City University, 22-2 Seto, Kanazawa-ku, Yokohama 236-0027, Japan.

* Correspondence: Tadaaki Nakajima, Department of Science, Yokohama City University, 22-2 Seto, Kanazawa-ku, Yokohama 236-0027, Japan, (nakajima.tad.uu@yokohama-cu.ac.jp).

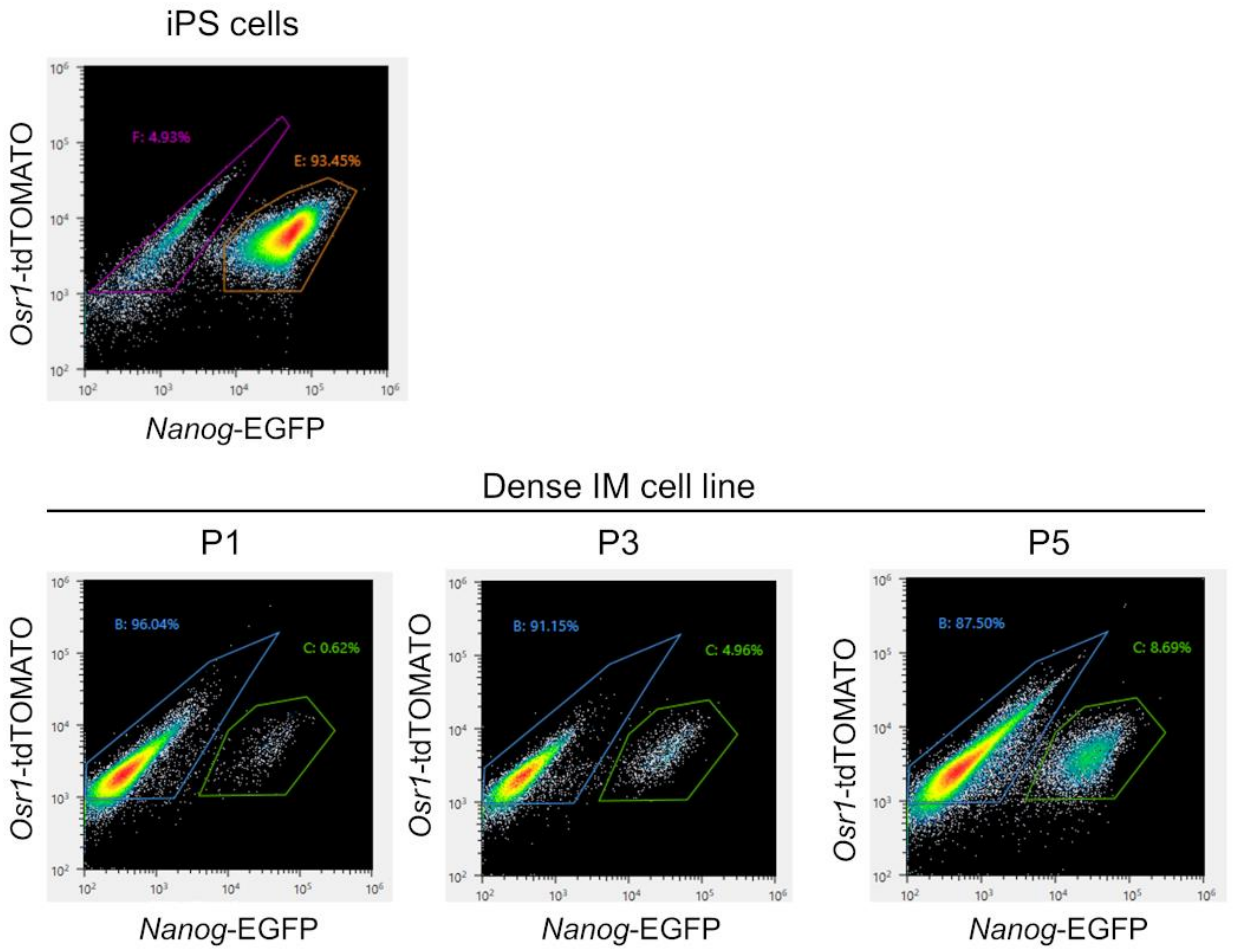

Figure S1. The population of pluripotent and differentiated cells in the IM cell line. In dense IM cell lines from passage number 1 (P1) to P5 and iPS cells, *Nanog*-EGFP and *Osr1*-tdTOMATO were detected by FACS (n=5).

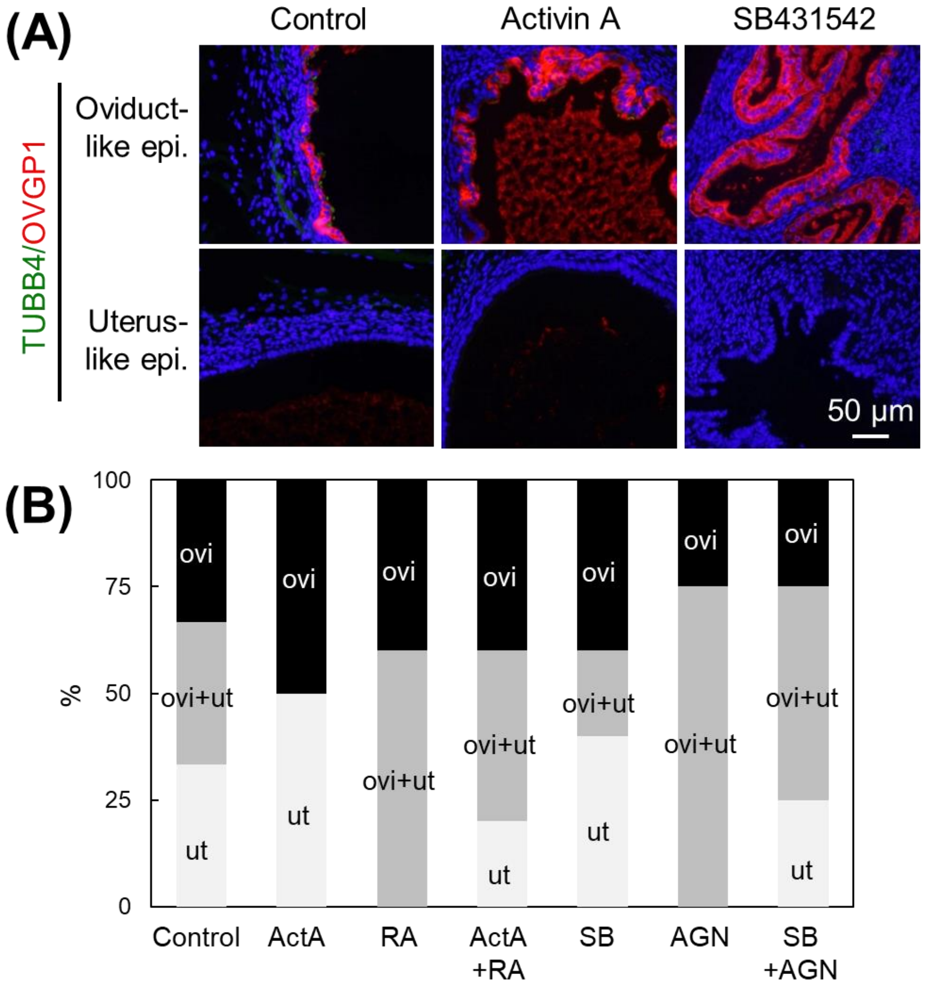

Figure S2. The effect of activation of SMAD2/3 signaling in MD at E13.5. E13.5 MDs were cultured with activin A (ActA), SB431542 (SB), RA, and/or AGN193109 (AGN) for 7 days, and organ-cultured MDs were grafted for 30 days. (A): TUBB4 (oviductal ciliated epithelial marker) and OVGP1 (oviductal secretory epithelial marker) expression in grafted MDs (n=4-6). Scale bar=50 μm. (B): The rate of number of ducts having oviduct-like epithelium (ovi) containing the TUBB4 and/or OVGP1-expressing cells, uterus-like epithelium (ut) consisting of TUBB4- and OVGP1-negative cells, and both (ovi+ut) in grafted MDs.

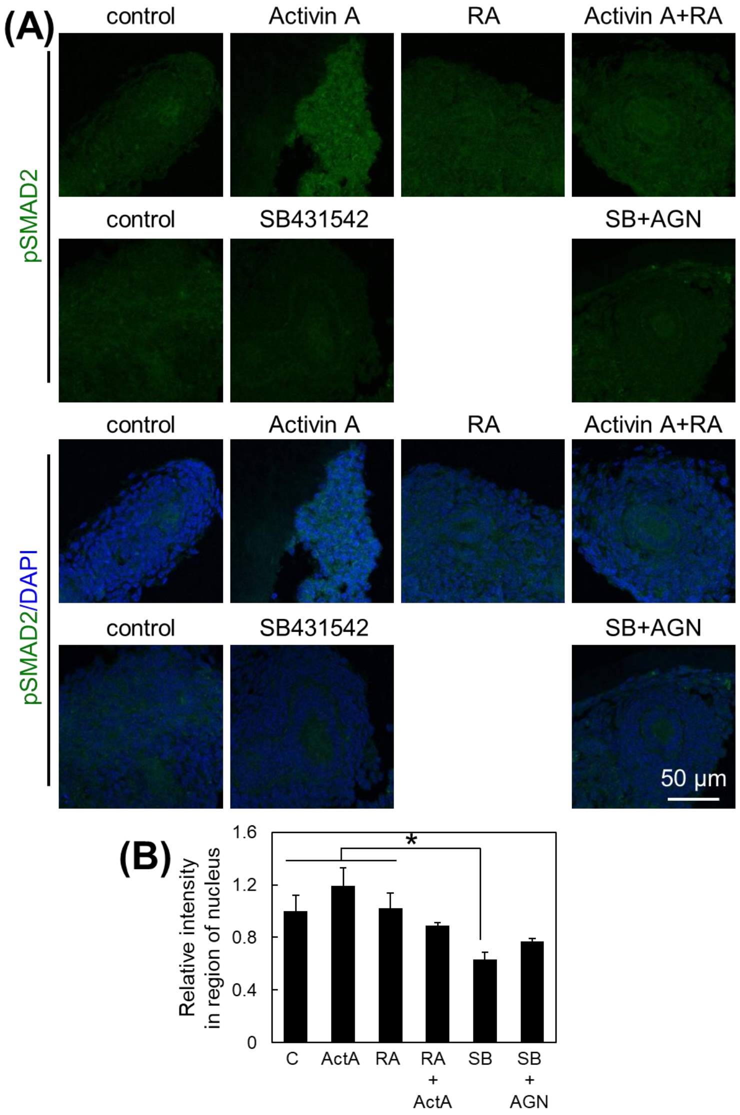

Figure S3. SMAD2 activation in organ-cultured MDs. (A): Localization of pSMAD2 and merged images with DAPI staining in organ-cultured E11.5 MDs. E11.5 MDs were cultured with activin A, SB431542 (SB), RA, and/or AGN193109 (AGN) for 7 days (n=3-5). Green: pSMAD2-positive cells. Blue: nuclei. Scale bar=50 μm. (B): pSMAD2 intensity in the nucleus was quantified in DAPI-positive nuclei of organ-cultured MDs. *: $p \leq 0.05$ by Games-Howell test. The error bars represent SEM.

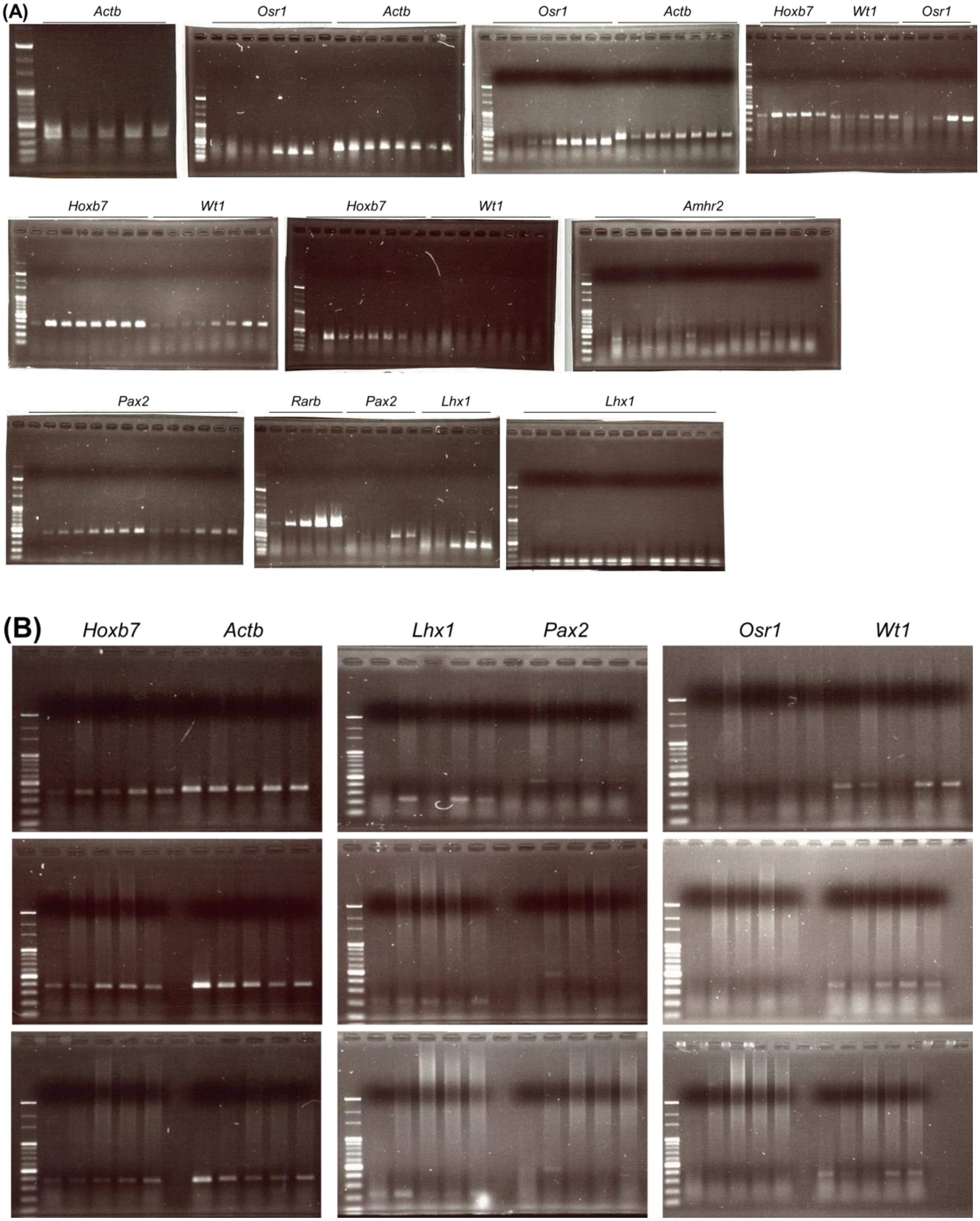
(A)
Actb
Osr1
Actb
Osr1
Actb
Hoxb7
Wt1
Osr1
Hoxb7
Wt1
Hoxb7
Wt1
Amhr2
Pax2
Rarb
Pax2
Lhx1
Lhx1
(B)
Hoxb7
Actb
Lhx1
Pax2
Osr1
Wt1

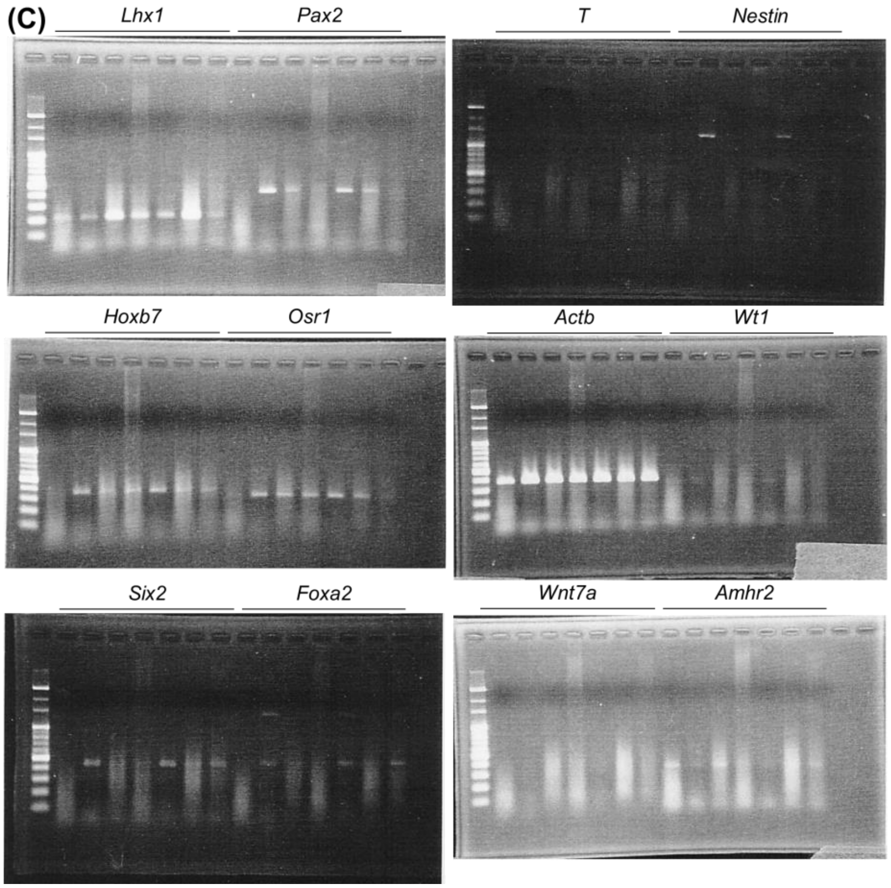

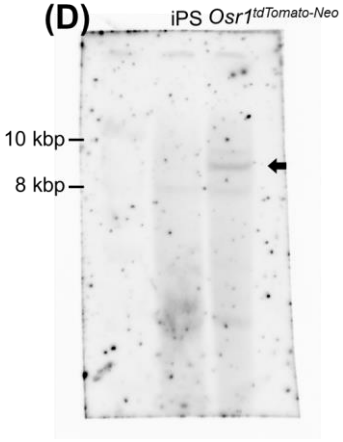

Figure S4. The raw gel images. (A): The gel images of RT-PCR with 100 bp marker for Fig 2C and D. (B): The gel images of RT-PCR with 100 bp marker for Fig 2E. (C): The gel images of RT-PCR with 100 bp marker for Fig 3C. (D): The image of Southern blot for Fig 3E.

Table S1. Primers for homologous recombination.

Gene	Forward sequence (5’→3’)	Reverse sequence (5’→3’)
5’ arm of *Osr1*	GGGGACAACTTGTATAGAAAAGTTGGACCTCGGGAGGAAGGAAGGAGACA	GGGGACTGCTTTTTTGTACAAACTTGAGTACTCGAGGCGCCACCGGTTCTGTCGCTGGGAACCGCAATGAT
3’ arm of *Osr1*	GGGGACAGCTTTCTTGTACAAAGTGGTTCTAAAGTGCCAGGTGCGGTGTCC	GGGGACAACTTTGTATAATAAAGTTGTGTCAGGGGCTGAAGCCAGAAAAGC
Pgk-Neo probe	GGCATTCTGCACGCTTCAAA	TCAGAAGAACTCGTCAAGAA

Table S2. Primers for RT-PCR

Gene	Forward sequence (5’→3’)	Reverse sequence (5’→3’)	Cycle
Lhx1	AACGACTTCTTCCGATGTTTCG	TTGGCGACACTGCTGTTACT	30
Pax2	AAACGCGAGGAAGATGTGTCT	GATGGCTGTATGGGTTGCCT	28
Osr1	AGGGATGAGTGAGACGTAGC	CCATTTCTGTCGCTGGGAAC	30
Wt1	TCCGAGGCATTCAGGATGTG	ATGAGTCCTGGTGTGGGTCT	23
Hoxb7	GCTCGAACCGAGTTCCTTCA	TTCCCGGTCCTGAGGTTTTG	28
Six2	GAGGCCAAGGAAAGGTACGA	CCTCTCTGAAACTCGCCGTT	28
Wnt7a	TTGCGCTTGTTCTCCCCTC	CCGAAGAGAAGCCACCGAT	32
Amhr2	GTGCCCTGTATTTGTAGGAAGC	TCTCGGCATCCTTGCATCTC	32
Foxa2	GTATGCTGGGAGCCGTGAA	GTGTAGCTGCGTCGGTATGT	30
T	CTCTCCAACCTATGCGGACAA	CCGTGTCATACTGGCTGTCA	30
Nestin	AGCTGGAGCGCGAGTTAGA	ACCTTCCAGGATCTGAGCGA	30
Tgfb1	AGCTGCGCTTGCAGAGATTA	AGCCCTGTATTCCGTCTCCT	-
Tgfb2	CCTCCGAAAATGCCATCCC	CACTCTGGCTTTGGGGTTTTG	-
Tgfb3	ATGACCCACGTCCCCTATCA	CAGACGGCCAGTTCATTGTG	-
Inha	TCTGTCTCCTCGGTCCTGT	TCACAGGTGGCACAGGGTA	-
Inhba	CAGTAGTGGAGCGTGCAGAA	GCACGTCCAGGGAACTCTTT	-
Inhbb	TCAGCTTTGCAGAGACAGATGG	GTCTCCGTGACCCTGTTCTT	-
Fst	TGCAACTCCATCTCGGAAGAA	GCCCAAAGGCTATGTCAACAC	-
Nodal	ACATCCAGAGCCTGCTGAAAC	CACCCACACTCCTCCACAAT	-
Actb	TGTTACCAACTGGGACGACA	TCTCAGCTGTGGTGGTGAAG	-
